# The interplay between *Campylobacter* and *Helicobacter* species and other gastrointestinal microbiota of commercial broiler chickens

**DOI:** 10.1186/1757-4749-6-18

**Published:** 2014-06-04

**Authors:** Nadeem O Kaakoush, Nidhi Sodhi, Jeremy W Chenu, Julian M Cox, Stephen M Riordan, Hazel M Mitchell

**Affiliations:** 1 School of Biotechnology and Biomolecular Sciences, The University of New South Wales, Sydney, NSW 2052, Australia; 2 Birling Avian Laboratories, Bringelly, NSW 2556, Australia; 3 Faculty of Science, The University of New South Wales, Sydney, NSW 2052, Australia; 4 Gastrointestinal and Liver Unit, The Prince of Wales Hospital, Randwick, NSW 2031, Australia; 5 Prince of Wales Clinical School, The University of New South Wales, Sydney, NSW 2052, Australia

**Keywords:** Broiler chicken, Gastrointestinal tract, Microbiota, Pathogen, *Campylobacter*, *Helicobacter*, *Gallibacterium*, *Campylobacter concisus*

## Abstract

**Background:**

Poultry represent an important source of foodborne enteropathogens, in particular thermophilic *Campylobacter* species. Many of these organisms colonize the intestinal tract of broiler chickens as harmless commensals, and therefore, often remain undetected prior to slaughter. The exact reasons for the lack of clinical disease are unknown, but analysis of the gastrointestinal microbiota of broiler chickens may improve our understanding of the microbial interactions with the host.

**Methods:**

In this study, the fecal microbiota of 31 market-age (56-day old) broiler chickens, from two different farms, was analyzed using high throughput sequencing. The samples were then screened for two emerging human pathogens, *Campylobacter concisus* and *Helicobacter pullorum*, using species-specific PCR.

**Results:**

The gastrointestinal microbiota of chickens was classified into four potential enterotypes, similar to that of humans, where three enterotypes have been identified. The results indicated that variations between farms may have contributed to differences in the microbiota, though each of the four enterotypes were found in both farms suggesting that these groupings did not occur by chance. In addition to the identification of *Campylobacter jejuni* subspecies *doylei* and the emerging species, *C. concisus*, *C. upsaliensis* and *H. pullorum*, several differences in the prevalence of human pathogens within these enterotypes were observed. Further analysis revealed microbial taxa with the potential to increase the likelihood of colonization by a number of these pathogens, including *C. jejuni*.

**Conclusion:**

Depletion of these taxa and the addition of taxa that compete with these pathogens, may form the basis of competitive exclusion strategies to eliminate them from the gastrointestinal tract of chickens.

## Background

Contaminated poultry products are a major source of human foodborne acute bacterial gastroenteritis pathogens worldwide [1]. Epidemiological studies indicate that between 50-80% of all human *Campylobacter* infections are attributed to poultry, in particular the handling and consumption of raw or undercooked broiler chicken meat [1]. Given that the majority of warm-blooded domestic animals as well as wild animals and birds shed viable *Campylobacter* species in their feces, it is not surprising that these organisms are abundant on poultry farms and in the surrounding environment [2]. Colonization of broiler flocks with *Campylobacter* species typically occurs between 2–3 weeks of age and *Campylobacter*-positive birds often remain colonized until slaughter [3]. Moreover, although high levels (up to 10^9^ bacteria/g) of *Campylobacter* spp. have been recovered from the ceca, chickens appear to suffer no clinical or other adverse effects [3]. More recently, other emerging *Campylobacter* and the closely related *Helicobacter* species have been detected within the microbiota of chickens and on processed chicken meat [4,5], suggesting that chickens may also serve as a reservoir for these species, and thus, may also be transmitted to humans.

The annual costs associated with campylobacteriosis are extremely high, estimated to be $1.7 billion in the United States alone [6]. Given the association between chickens and campylobacteriosis, as well as the high costs associated with this disease, many countries have investigated intervention strategies to reduce or eliminate *Campylobacter* from the chicken meat primary production and processing chain [7]. Such intervention strategies not only address the spread of *Campylobacter* species on farms and the surrounding environment, but also strategies aimed at reducing the bacterial load of *Campylobacter* spp. in the intestinal tract of infected chickens or increasing the resistance of chickens to *Campylobacter* carriage [3,8].

While intervention strategies are currently being employed to limit the transmission of *Campylobacter* species on farms and their surrounding environment, adherence to these strategies differs considerably among farms. Based on the current literature the use of hygiene barriers at the entrance to poultry houses, the provision of hand washing facilities, boot dips, house specific boots and overshoes have all been shown to prevent *Campylobacter* colonization of chicks [2,3,9]. Other strategies that have been developed to combat the bacterial load of *Campylobacter* spp. in the intestinal tract of infected chickens include vaccination and the use of bacteriocins, bacteriophages and probiotics [10,11]. Such approaches have been reported to lead to a reduction in intestinal colonization levels of broiler chickens and in some cases have resulted in a considerable decline in human campylobacteriosis rates [12]. Indeed, a quantitative microbial risk assessment determined that a reduction of 2.0 log_10_*Campylobacter* cells per broiler carcass would result in a 30-fold decline in human campylobacteriosis [13].

In some countries, competitive exclusion has been successfully employed to limit the colonization of chickens with *Salmonella* and *Escherichia coli*[14,15]. Potential competitive exclusion strategies to limit the prevalence of *C. jejuni* have also been investigated, with some degree of success [16-18]. Given this, further insights into the microbiota of chickens may aid the development of competitive exclusion strategies for *Campylobacter* and *Helicobacter* species. Thus, in the current study, the fecal microbiota of 31 market-age broiler chickens were analyzed using high-throughput sequencing and PCR to determine the prevalence and relative abundance of *Campylobacter* and *Helicobacter* species, and to identify bacterial taxa that may be associated with the absence or carriage of these enteric pathogens in commercial poultry.

## Results and discussion

### Classification of the gastrointestinal microbiota of chickens

**Figure 1:**
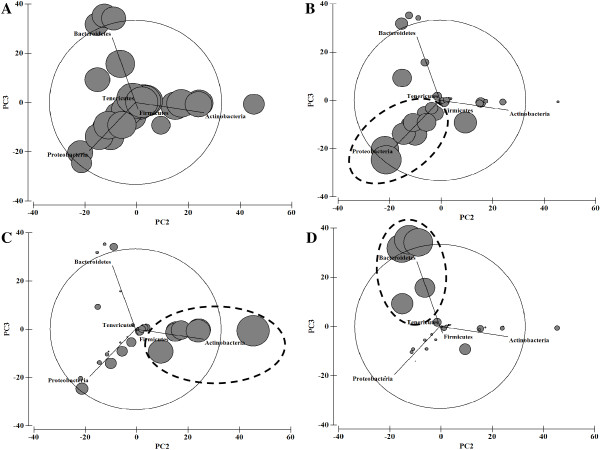
**Principal component analysis of the 31 chicken microbiota samples.** Circles reflect the abundance of Firmicutes **(A)**, Proteobacteria **(B)**, Actinobacteria **(C)**, and Bacteroidetes **(D)**. Dotted lines encompass samples differentiated into enterotype 2 **(B)**, 3 **(C)** and 4 **(D)**. Enterotype 1 samples aggregate in the center and cannot be differentiated along these axes.

The gastrointestinal microbiota plays an important role in the growth and development of chickens [19]. Several important human pathogens are commonly found within the chicken microbiota, though typically they are non-pathogenic to chickens [3]. As a result, chickens are one of the key reservoirs for transmission of foodborne disease. To gain a better understanding of the influence of the chicken gastrointestinal microbiota on the carriage of *Campylobacter* and *Helicobacter* species, the fecal microbiota of 31, 56-day old chickens originating from two different farms were analyzed using high throughput sequencing (average number of reads ± SEM: 18227 ± 1836). Based on PCA, the microbiota were separated into four potential enterotypes: enterotype 1 dominated by Firmicutes, enterotype 2 by Firmicutes and Proteobacteria, enterotype 3 by Firmicutes and Actinobacteria and enterotype 4 by Firmicutes and Bacteroidetes (Figure 1). Analysis using SIMPER confirmed these groupings with the cumulative contribution of these taxa being higher than 95% for each of these enterotypes (Table 1).

**Table 1:** SIMPER analysis of the cumulative contribution of taxa to the overall microbiota of the enterotype

**Enterotype**	**Phylum**	**Average abundance**	**Average similarity**	**Similarity/SD**	**Contribution (%)**	**Cumulative contribution (%)**
1	Firmicutes	95.23	93.69	32.17	97.50	97.50
2	Firmicutes	71.24	62.53	4.34	77.02	77.02
	Proteobacteria	24.97	17.41	2.26	21.45	98.47
3	Firmicutes	61.09	51.09	3.01	66.15	66.15
	Actinobacteria	31.34	24.43	5.23	31.64	97.79
4	Firmicutes	58.44	53.78	22.41	64.76	64.96
	Bacteroidetes	31.84	25.72	2.69	30.96	95.72

Percentage cut-off for contributing taxa was 95%.

**Figure 2:**
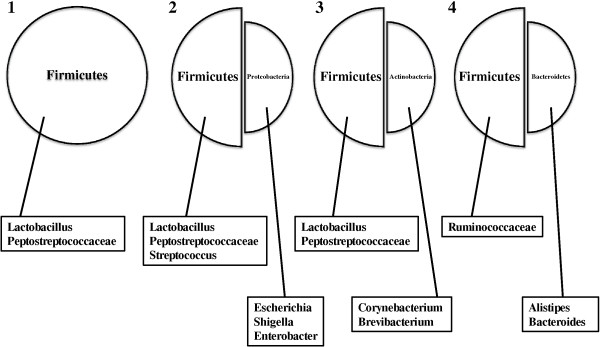
Taxa identified to be the highest contributors within each enterotype.

Upon further analysis, it is possible that enterotypes 2 and 3 were derived from enterotype 1, with the dominant Firmicutes being *Lactobacillus* and *Peptostreptococcaceae* for all three groupings (Figure 2). Conceivably, chickens with microbiota classified as enterotype 1 were at some later point efficiently colonized by either Proteobacteria (enterotype 2) or Actinobacteria (enterotype 3), thus resulting in the shared dominance between the respective taxa. However, only studies investigating the development of the microbiota during rearing will definitively ascertain the evolution of these enterotypes. In contrast, the Firmicutes from enterotype 4 were dominated by *Ruminococcaceae*, and this dominance was shared with the Bacteroidetes taxa *Alistipes* and *Bacteroides* (Figure 2). One explanation for this is that colonization by Bacteroidetes resulted in a shift in dominance from *Lactobacillus* and *Peptostreptococcaceae* to *Ruminococcaceae*, as these bacterial groups were present in enterotypes 1, 2 and 3, albeit in lower abundance. In addition to these findings, the lack of differentiation of dominant taxa beyond the family classification suggests that, in some cases, novel taxa may be present, as has been observed previously in the gut microbiota of post-hatch broiler chickens [20]. Interestingly, the dominance of Firmicutes, in particular *Lactobacillus*, within the gastrointestinal microbiome of broiler chickens has been documented previously [21-24]. However, to our knowledge, the possible grouping of the chicken microbiota into four different enterotypes and the differences in their composition has not been previously reported.

To investigate these enterotype groupings further, the microbial diversity within the 31 samples was examined. Enterotypes 1, 2 and 4 had a similar average number of species and species richness, and this was significantly lower than that of enterotype 3 (ANOVA, *P* < 0.005) (Table 2). The diversity (Shannon index, H’) of enterotypes 3 and 4 were the highest among the enterotypes, reflecting the high species richness in enterotype 3 (Table 2), the higher contribution of the non-Firmicutes taxa within both of these enterotypes (Table 1) and the different composition of Firmicutes within enterotype 4 (Figure 2). In contrast, the diversity for enterotype 1 was significantly lower than the other three enterotypes (*P* < 0.0001), most probably due to the extensive dominance of Firmicutes.

**Table 2:** Microbial diversity and pathogen prevalence in 31 chicken samples determined using pyrosequencing

**Enterotype**	**Sample**	**Farm**	**Species**	**Species richness**	**H’**	**Cjj**	**Cjd**	**Cco**	**Cup**	**Hpu**	**Hbr**	**Gal**
1	1	1	169	36.50	2.248	-	+	+	-	-	+	+
	13	2	150	17.60	2.309	-	-	-	-	-	-	-
	16	2	85	18.24	1.185	-	-	-	-	-	-	-
	23	2	132	28.45	1.874	+	+	-	+	-	+	+
	24	2	127	27.36	2.350	-	-	-	-	-	-	+
	25	2	104	22.37	1.661	+	+	-	-	-	+	+
	27	2	87	18.67	2.053	-	-	-	-	-	-	-
	28	2	133	28.66	1.506	+	+	-	+	+	+	+
	29	2	118	25.41	2.282	+	+	-	-	-	+	+
Average			123	24.81	1.940							
Prevalence						44.4	55.6	11.1	22.2	11.1	55.6	66.7
2	4	1	97	20.85	2.049	+	+	-	+	-	+	+
	6	1	113	24.82	2.970	+	+	-	-	-	+	-
	8	1	200	43.21	2.701	+	+	-	-	+	+	+
	9	1	139	29.97	2.987	+	-	-	-	-	+	+
	10	1	23	4.78	2.490	-	-	-	-	-	-	-
	17	2	102	21.93	2.413	-	+	-	-	-	+	+
	20	2	71	15.20	1.659	-	-	-	-	-	-	-
	21	2	107	23.02	1.598	-	+	-	-	+	+	+
	26	2	92	19.76	2.434	-	+	-	-	+	+	+
	31	2	162	34.96	2.632	+	+	+	-	-	+	+
Average			111	23.85	2.390							
Prevalence						50.0	70.0	10.0	10.0	30.0	80.0	70.0
3	2	1	186	40.17	3.244	-	-	-	-	-	-	-
	11	2	129	27.79	2.327	-	-	-	-	-	-	+
	14	2	156	33.66	2.751	+	+	-	-	-	+	+
	15	2	130	28.01	2.518	-	-	-	-	-	-	+
	18	2	167	36.05	2.758	-	-	-	-	-	-	-
	19	2	213	46.04	3.236	-	+	-	-	-	+	+
	30	2	185	39.96	3.791	+	+	-	-	-	+	-
Average			167	35.95	2.950							
Prevalence						28.6	42.9	0	0	0	42.9	57.1
4	3	1	91	19.54	2.570	+	+	-	-	-	+	-
	5	1	112	24.10	2.830	+	+	-	-	+	+	-
	7	1	140	30.18	3.059	-	+	-	-	+	+	-
	12	2	150	32.35	3.015	-	+	-	-	+	+	-
	22	2	46	9.77	2.835	-	-	-	-	-	-	-
Average			108	23.19	2.860							
Prevalence						40.0	80.0	0	0	60.0	80.0	0
Total prevalence						41.9	61.3	6.4	9.7	22.6	64.5	54.8

H’ is the Shannon index; Cjj is *C. jejuni* subsp. *jejuni*, Cjd is *C. jejuni* subsp. *doylei*; Cco is *C. concisus*; Cup is *C. upsaliensis*; Hpu is *Helicobacter pullorum*; Hbr is *Helicobacter brantae*; and Gal: *Gallibacterium anatis*.

### Effect of farming practices on broiler chicken gastrointestinal microbiota

Both broiler farms used in the study belong to the same vertically integrated commercial broiler chicken operation and are located within a 25 km radius of each other. Farm 1 operates as a conventional (indoor barn) housing system, whereas farm 2 practices a free-range (extensive) system. The key differences between the two management practices is that free-range flocks are allowed access to outdoor areas, are stocked at lower densities (16–32 kg/m^2^ vs 28–40 kg/m^2^), and the therapeutic and/or prophylactic use of antibiotics is prohibited. Samples collected from both broiler farms were found to contain all four enterotypes (Table 2). Interestingly, no significant differences in the number of species (Farm 1: 127 vs. Farm 2: 126, *P* = 0.95), species richness (Farm 1: 27.4 vs. Farm 2: 26.4, *P* = 0.79) or species diversity (Farm 1: 2.715 vs. Farm 2: 2.342, *P* = 0.095) were found between farms; however, chickens from farm 1 appeared to be predominantly classified under enterotype 2 and 4, reflecting an increased dominance of Gram-negative bacteria. Although the number of samples analyzed from farm 1 was low and our knowledge of the identity and quantity of the administered antibiotics limited, these findings raise the possibility that farming practices, particularly antibiotic use, may influence the identity of the taxa within the gastrointestinal microbiota of broiler chickens, but not the species richness. These findings are of particular interest given that Torok and colleagues have reported an OTU assigned to *Enterobacteriaceae* to be less prevalent in the gut of chicks raised on an antimicrobial-free diet and OTUs assigned to a range of Gram-positive Firmicutes, including *Lachnospiraceae*, *Lactobacillus johnsonii*, *Ruminococcaceae* and *Oxalobacteraceae*, to be less prevalent in the guts of chicks fed antimicrobial-supplemented diets [20].

### Differences between the chicken and human gastrointestinal microbiota

Based on our own findings and those of others [25,26], there is a clear difference in the composition of the chicken and human gastrointestinal microbiota. In chickens the highest contributors have been identified as *Lactobacillus* (average abundance = 35.05%) and *Peptostreptococcaceae* (average abundance = 19.48%). However, in humans, while Firmicutes also dominate the microbiota, the taxa identified to play a major role include *Blautia*, *Roseburia*, *Faecalibacterium*, *Ruminococcus*, *Coprococcus*, *Clostridium*, *Subdoligranulum* and *Bacteroides* (Table 3) [27,28]. The importance of these differences requires further investigation, as potentially such differences, in addition to other physiological factors such as core body temperature and cell surface receptors, may explain why chickens infected with *Campylobacter* and other human pathogens do not develop clinical disease.

**Table 3:** SIMPER analysis of the cumulative contribution of taxa to the overall microbiota of the human gastrointestinal tract

**Genus**	**Average abundance**	**Average similarity**	**Similarity/SD**	**Contribution (%)**	**Cumulative contribution (%)**
*Blautia*	15.68	9.51	2.02	17.44	17.44
*Roseburia*	12.76	9.12	2.22	16.72	34.16
*Faecalibacterium*	13.65	7.76	1.16	14.22	48.37
*Ruminococcus*	9.72	5.6	1.45	10.26	58.63
*Coprococcus*	7.08	5.54	2.99	10.15	68.78
*Clostridium*	6.84	5.13	2.78	9.41	78.19
*Subdoligranulum*	5.53	4.06	2.61	7.44	85.63
*Bacteroides*	8.91	3.03	0.57	5.55	91.18

Results were obtained from the analysis of 20 healthy controls previously analyzed by our group [27]. Percentage cut-off for contributing taxa was 90%.

### Prevalence of *Campylobacter, Helicobacter* and *Gallibacterium* species in chickens

The prevalence of *Campylobacter*, *Helicobacter* and *Gallibacterium* species were also determined in the 31 chicken samples using the pyrosequencing data (Table 2). This showed that *C. jejuni* subsp. *jejuni* was detected in 41.9%, *Campylobacter jejuni* subsp. *doylei* in 61.3%, *C. concisus* in 6.4%, *Campylobacter upsaliensis* in 9.7%, *H. pullorum* in 22.6%, *Helicobacter brantae* in 64.5% and *Gallibacterium anatis* in 54.8% of chicken samples (Table 2). Given that pyrosequencing is less sensitive than species-specific PCR, it is likely that this approach may underestimate the prevalence of some of these species.

The prevalence of both *C. jejuni* subspecies were observed to be lower in enterotype 3 than in the other enterotypes, suggesting that Actinobacteria may compete with these bacteria within the gastrointestinal tract. Similarly, the dominance of Bacteroidetes appeared to have a negative effect on *G. anatis*, as this bacterium was absent in enterotype 4. To confirm these trends, further studies using a higher number of samples from all enterotypes should be analyzed and the application of species-specific PCR performed. *C. jejuni* subsp. *doylei* was detected at high prevalence in fecal samples from these chickens. This bacterium causes both gastritis and enteritis; however, it is more commonly isolated from blood cultures than stool cultures from human patients [29]. Interestingly, over a five-year period, *C. jejuni* subsp. *doylei* was isolated from 85.2% of *Campylobacter*/*Helicobacter*-related bacteremia cases in Australia [29].

In the current study, *H. brantae* was also detected at high prevalence in these broiler fecal specimens. This urease-negative *Helicobacter* was first isolated from Canada geese within the greater Boston area [30]; however, little is known about the pathogenesis of this bacterium. In addition to the above species, two emerging pathogens *C. concisus* and *C. upsaliensis*[31,32] were detected at low prevalence in these chickens (Table 2), suggesting that chickens may act as a reservoir of emerging *Campylobacter* species. Lynch *et al.* have previously reported the detection and isolation of these fastidious *Campylobacter* species from chicken meat [4], indicating that, similar to the closely related *C. jejuni*, these species can remain viable following processing.

### Influence of *Campylobacter, Helicobacter* and *Gallibacterium* on the composition of the chicken gastrointestinal microbiota

The microbial composition of the chicken gastrointestinal tract was then analyzed with respect to the presence of these bacteria, independent of the enterotype of the chickens. Correlation analysis was performed between each of the genera *Campylobacter*, *Helicobacter* and *Gallibacterium* against all other genera detected in these samples. This showed that the abundance of *Helicobacter* correlated with that of *Ureaplasma* (*r* = 0.58, 95% CI: 0.28-0.77, *P* = 0.0006), though the significance of this correlation is unclear.

We then employed SIMPER to determine the effect of these pathogens on other taxa within the microbiota (Additional file 1). For each pathogen we identified the taxa that adhered to the following four conditions: 1) Presence within the 95% contribution cut-off, 2) contribution to the similarity between the positive samples, 3) contribution to the dissimilarity between the positive and negative samples, and 4) the lack of contribution to the similarity of the negative samples. For *C. jejuni*, these taxa were *Escherichia*, *Alistipes*, *Enterococcus*, *Bacteroides*, *Shigella*, *Gallibacterium*, *Campylobacter*, *Faecalibacterium*, *Blautia*, *Enterobacter* and *Clostridium*. Moreover, the presence of *C. jejuni* was also associated with a lower abundance of *Lactobacillus* (32.2% vs. 40.3%) and *Corynebacterium* (2.0% vs. 8.9%) and a higher abundance of both *Streptococcus* (4.4% vs. 1.9%) and *Ruminococcaceae* (5.8% vs. 3.3%). Interestingly, Bereswill *et al.* have recently reported that, in humans, a diet-induced alteration of the intestinal microbiota, comprising an increase in the abundance of *E. coli* and *Eubacterium* spp. and a decrease in *Enterococcus* and *Lactobacillus* spp., was associated with a greater susceptibility to *C. jejuni* infection [33]. This is in line with our findings that showed that *Lactobacillus* spp. were lower in abundance and *Escherichia* was a major contributor in chickens colonized with *C. jejuni*. Moreover, three other identified taxa, *Bacteroides*, *Alistipes* and *Blautia*, are all major producers of short-chain fatty acids (SCFA). Given that *C. jejuni* can utilize acetate and lactate as carbon sources and these SCFAs contribute to *C. jejuni* colonization of the gut [34], it is possible that SCFAs produced by these genera could potentially serve as energy sources for *C. jejuni* and influence its ability to colonize the chicken gastrointestinal tract. In addition to this, four of the taxa (*Faecalibacterium*, *Blautia*, *Clostridium* and *Bacteroides*) that have been found to be major contributors to the human gastrointestinal microbiota were also associated with the presence of *C. jejuni* in the chicken gastrointestinal tract.

Similarly, SIMPER analysis for *H. pullorum* identified *Bacteroides*, *Alistipes*, *Faecalibacterium*, *Coprococcus*, *Blautia*, *Gallibacterium*, *Clostridium*, *Pseudobutyrivibrio*, *Sutterella*, *Megamonas*, *Roseburia*, *Campylobacter*, *Anaerotruncus*, *Helicobacter* and *Barnesiella* to be taxa of interest. As observed with *C. jejuni*, the presence of *H. pullorum* was associated with a lower abundance of *Lactobacillus* (21.8% vs. 38.9%) and *Corynebacterium* (0.66% vs. 5.5%) and a higher abundance of *Streptococcus* (7.6% vs. 2.3%) and *Ruminococcaceae* (9.3% vs. 3.6%). In contrast, *Gallibacterium* was the only taxon identified to satisfy the four conditions for the *H. brantae* positive samples. In addition, *H. brantae* was associated with a lower abundance of *Ruminococcaceae* (2.6% vs. 8.1%) and higher abundance of *Peptostreptococcaceae* (22.8% vs. 14.9%) and *Corynebacterium* (6.4% vs. 1.7%).

No taxa were identified that adhered to the four conditions for either *C. upsaliensis* or *G. anatis*, though the presence of either of these taxa was associated with substantially higher levels of *Lactobacillus* (51.2% vs. 33.3% and 42.5% vs. 26.0%, respectively) and *Peptostreptococcaceae* (38.0% vs. 17.5% and 25.6% vs. 12.1%, respectively). Such differences may reflect the effect of these bacterial species on the host microbiota, or alternatively, their growth requirements and ability to compete for nutrients within the gastrointestinal tract.

### Prevalence of *Campylobacter concisus* and *Helicobacter pullorum* in chickens

To date there have been a limited number of studies on the prevalence of emerging *Campylobacter* and *Helicobacter* species in chickens [4,5]. Given that *C. concisus* and *H. pullorum* were identified in these chickens, the prevalence of these bacteria using a more sensitive approach (species-specific PCR) was further investigated. Based on PCR, *C. concisus* and *H. pullorum* were shown to be present in 22.6% and 32.2% of chicken samples, respectively (Table 4), confirming that chickens have the potential to act as reservoirs for these species.

**Table 4:** **Prevalence of *Campylobacter concisus *** **and *Helicobacter pullorum *** **in 31 chicken samples determined using pyrosequencing and PCR**

**Enterotype**	**Sample**	**Farm**	**Cco**	**Hpu**
1	1	1	+	+
	13	2	-	-
	16	2	-	-
	23	2	+	-
	24	2	-	+
	25	2	+	-
	27	2	-	-
	28	2	-	+
	29	2	+	-
Prevalence			44.4	33.3
2	4	1	-	-
	6	1	-	-
	8	1	-	+
	9	1	-	-
	10	1	-	-
	17	2	-	-
	20	2	-	-
	21	2	-	+
	26	2	+	+
	31	2	+	-
Prevalence			20.0	30.0
3	2	1	-	-
	11	2	-	+
	14	2	-	-
	15	2	-	-
	18	2	-	-
	19	2	-	-
	30	2	+	-
Prevalence			14.3	14.3
4	3	1	-	-
	5	1	-	+
	7	1	-	+
	12	2	-	+
	22	2	-	-
Prevalence			0	60.0
Total prevalence			22.6	32.2

Cco is *C. concisus*; and Hpu is *Helicobacter pullorum*.

SIMPER analysis revealed *Campylobacter* to be the only taxon of interest in *C. concisus*-positive samples (Additional file 2). However, similar to *C. upsaliensis*, *C. concisus* was associated with higher levels of *Lactobacillus* (44.0% vs. 32.4%) and *Peptostreptococcaceae* (29.1% vs. 16.7%), suggesting that these emerging *Campylobacter* species are more prevalent in a gastrointestinal tract substantially dominated by Firmicutes.

SIMPER analysis showed *H. pullorum* prevalence to be associated with similar taxa to those previously identified, including *Bacteroides*, *Alistipes*, *Faecalibacterium*, *Coprococcus*, *Blautia*, *Clostridium*, *Gallibacterium*, *Pseudobutyrivibrio*, *Sutterella*, *Atopostipes* and *Megamonas* (Additional file 2). Similar to the previous analysis, the presence of *H. pullorum* was associated with a lower abundance of *Lactobacillus* (30.1% vs. 37.4%) and a higher abundance of *Streptococcus* (6.0% vs. 2.4%) and *Ruminococcaceae* (7.0% vs. 3.9%). In contrast, in the new analysis, the prevalence of *Corynebacterium* was found to be higher in the *H. pullorum*-positive samples (6.2% vs. 3.6%).

## Conclusions

In the current study we have shown that the gastrointestinal microbiota of chickens could be classified into potential enterotypes, which is consistent with the detection of three enterotypes in humans [35]. While variations between farms may have contributed to differences in prevalence of specific enterotypes, the identification of each of the enterotypes on both farms suggests that these groupings were not coincidental. A number of differences in the prevalence of *Campylobacter* and *Helicobacter* species within these enterotypes were found, which provides possible insights into the microbial taxa that may increase the likelihood of colonization by these pathogens. Depletion of these taxa and addition of competitive taxa has the potential to aid the development of competitive exclusion strategies to eliminate these pathogens from the gastrointestinal tract of chickens. Further studies assessing larger sample numbers of fecal specimens collected over time to account for fluctuations in the microbiota [36] and screening the lower and upper intestines and ceca of broiler chickens are required to confirm our findings.

## Methods

### Sampling and extraction protocol

Freshly voided chicken feces were aseptically collected into 70 ml fecal specimen containers (S5744F, Techno-Plas; St Marys, SA, Australia) from two broiler farms in the Sydney Basin region just prior to depopulation (flock age of 56 days). Samples were transported to the testing facility at 4°C and stored at -20°C until analysis. DNA was extracted using the method of Griffiths and colleagues [37]. The concentration and quality of DNA was measured using a Nanodrop ND-1000 Spectrophotometer (Nanodrop Technologies; Wilmington, USA).

### Tag-encoded FLX amplicon pyrosequencing

The microbial community was assessed by high-throughput sequencing of the 16S rRNA gene. Tag-encoded FLX amplicon pyrosequencing (bTEFAP) was performed as described previously [38-42] using the primers Gray28F (5′TTTGATCNTGGCTCAG) and Gray519r (5′ GTNTTACNGCGGCKGCTG) numbered in relation to *E. coli* 16S rRNA (variable regions 1–3). The sequence of the primers is not complementary to chicken DNA, thus preventing co-amplification of chicken sequences. Moreover, the primers span the variable region of the rRNA gene so that discrimination between closely related taxa can be performed. At the same time, the positioning of the primers allows for amplification of a large proportion of known 16S rRNA sequences. Generation of the sequencing library utilized a one-step PCR with a total of 30 cycles, a mixture of Hot Start and HotStar high fidelity *Taq* polymerases, and amplicons originating and sequencing extending from the 28 F position with an average read length of 400 bp. Tag-encoded FLX amplicon pyrosequencing analyses utilized a Roche 454 FLX instrument with Titanium reagents. This bTEFAP process was performed at the Molecular Research LP laboratory (MR DNA; Shallowater, TX) based upon established and validated protocols.

### Data analysis

The sequence data derived from the high-throughput sequencing process was analyzed employing a pipeline developed at Molecular Research LP. Sequences are first depleted of barcodes and primers, then short sequences (<200 bp), sequences with ambiguous base calls, and sequences with homopolymer runs exceeding 6 bp are all removed. Sequences were then de-noised and chimeras were removed (Black Box Chimera Check software B2C2) [43]. Operational taxonomic units (OTU) were defined after removal of singleton sequences (sequences appearing only once in the whole dataset) with clustering set at 3% divergence (97% similarity) [38-42]. OTUs were then taxonomically classified using BLASTn against a curated GreenGenes database [44] and compiled into each taxonomic level. Taxonomy was defined based on the following percentages: >97%, species; between 97% and 95%, unclassified species; between 95% and 90%, unclassified genus; between 90% and 85%, unclassified family; between 85% and 80%, unclassified order; between 80% and 77%, unclassified phylum; <77%, unclassified. Principal component analyses (PCA), diversity analyses (DIVERSE) and analysis of similarities and species contributions (SIMPER) based on relative abundances of bacterial groups were performed using Primer-E [45].

### Detection of *Campylobacter concisus* and *Helicobacter pullorum*

*Campylobacter concisus* DNA was amplified using a nested PCR procedure with the first step employing *Campylobacter* genus-specific primers, C412F and C1228R [46] and the thermal cycling conditions 94°C for 5 min, 40 cycles of 94°C for 10 s, 55°C for 10 s, and 72°C for 45 s, followed by 72°C for 7 min. The second amplification utilized the *C. concisus*-specific primers: 5′-CTT GTG AAA TCC TAT GGC TTA- 3′ (Concisus F) and 5′-CTC ATT AGA GTG CTC AGC C-3′ (Concisus R), which were previously optimized by Man *et al.*[47]. Cycling conditions were 40 cycles of 94°C for 10 s, 65°C for 10 s, and 72°C for 30 s. *Helicobacter pullorum* DNA was amplified using *H. pullorum*-specific primers targeting the 16S rDNA gene as previously described [48].

## Competing interests

No conflicts of interest exist.

## Authors’ contributions

HMM, JMC, JWC and NOK conceived the idea; HMM, SMR, JMC, JWC contributed reagents; JWC collected and organized all chicken samples; NS extracted and prepared all DNA samples; NOK performed the microbial community analyses; NS performed the species-specific PCRs; NOK, NS, HMM, JWC, JMC and SMR analysed the results; NOK, HMM, JWC, JMC and SMR drafted the manuscript. All authors read and approved the final manuscript.

## Supplementary Material

Additional file 1 **Contribution of taxa to the similarity and dissimilarity between subgroups of chicken.** Variables analyzed were the presence of *C. jejuni*, *C. upsaliensis*, *H. pullorum*, *H. brantae* and *G. anatis* derived from Table 2. The number of *C. concisus* positive samples was below the required threshold of 3 samples for statistical analysis. Percentage cut-off for contributing taxa was 95%. Click here for file

Additional file 2 **Contribution of taxa to the similarity and dissimilarity between subgroups of chicken.** Variables analyzed were the presence of *C. concisus* and *H. pullorum* derived from Table 3. Percentage cut-off for contributing taxa was 95%. Click here for file
